# Prediction and experimental evidence of different growth phases of the *Podospora anserina* hyphal network

**DOI:** 10.1038/s41598-023-35327-w

**Published:** 2023-05-25

**Authors:** Clara Ledoux, Florence Chapeland-Leclerc, Gwenaël Ruprich-Robert, Cécilia Bobée, Christophe Lalanne, Éric Herbert, Pascal David

**Affiliations:** Université Paris Cité, CNRS, UMR 8236 – LIED, 75013 Paris, France

**Keywords:** Fungal biology, Fungal evolution, Fungal physiology

## Abstract

Under ideal conditions, the growth of the mycelial network of a filamentous fungus is monotonous, showing an ever increasing complexity with time. The components of the network growth are very simple and based on two mechanisms: the elongation of each hypha, and their multiplication by successive branching. These two mechanisms are sufficient to produce a complex network, and could be localized only at the tips of hyphae. However, branching can be of two types, apical or lateral, depending on its location on the hyphae, therefore imposing the redistribution of the necessary material in the whole mycelium. From an evolutionary point of view, maintaining different branching processes, with additional energy needs for structure and metabolism, is intriguing. We propose in this work to discuss the advantages of each branching type using a new observable for the network growth, allowing us to compare growth configurations. For this purpose, we build on experimental observations of the *Podospora anserina* mycelium growth, enabling us to feed and constrain a lattice-free modeling of this network based on a binary tree. First, we report the set of statistics related to the branches of *P. anserina* that we have implemented into the model. Then, we build the density observable, allowing us to discuss the succession of growth phases. We predict that density over time is not monotonic, but shows a decay growth phase, clearly separated from an other one by a stationary phase. The time of appearance of this stable region appears to be driven solely by the growth rate. Finally, we show that density is an appropriate observable to differentiate growth stress.

## Introduction

The achievement of filamentous fungi in colonizing terrestrial ecosystems can be largely attributed to their flexible morphology, and more specifically to their ability to form an interconnected hyphal network, the mycelium. The growth of this structure is based upon some fundamental cellular processes, such as hyphal tip growth, septation, hyphal orientation, branching and fusion, also known as anastomosis^1^. Hyphal branching has been well described and appears to both increase the surface area of the colony, which enhances nutrient assimilation, as well as mediate hyphal fusion events that are important for the exchange of nutrients and signals within the mycelium^2^. Therefore, the architecture of the fungal network is clearly not fixed, but must continually adapt to local nutritional or environmental cues, damage or fungivore attacks. Such a network is constituted of apical branches (or leading hyphae), which are the first to invade new territory and are generally engaged in nutrient acquisition and sensing of the local environment, whereas behind the colony edge, subapical cells generate new hyphae by lateral branching^3^. An earlier description of such fungal network states that hyphal tips at the biomass edge are those associated with exploration, while hyphae behind the growth front are most associated with the exploitation of resources^4^.

All living systems are *a priori* motivated by the access to resources and reproduction, and constrained by its internal metabolism and the environment’s properties. Evolution proceeds from the selection of random mutations in the genotype that will retroact on the phenotype of the organism. This is the genotype-phenotype relationship as described in^5–7^ for different biological systems. This process seems to give rise to simple systems in the sense of Kolmogorov^8^, and this can be understood as a bias towards simplicity. Based on the algorithmic description of Kolmogorov’s complexity^9^, *i.e.* the size of the smallest program required to generate information, it is found that a short program is more likely to appear more frequently. Relying on this principle, it was recently suggested that simplicity and symmetry could emerge spontaneously from the evolutionary process^10^. The growth of a branching network can be most simply obtained by placing an active body at each tip, the Spitzenkörper (SPK) in the case of ascomycete filamentous fungi. This structure is believed to regulate the delivery of cell wall-building vesicles to the apical cell surface, thereby allowing the elongating of hyphae, as well as the generation of a new tip, *i.e.* a branching event^11–13^. However, the branching process is not observed only at the apexes, where apical branching occurs, but also in the subapical regions distant from the tip, virtually anywhere on the network. The latter is named lateral branching. This imposes the redistribution of the growth machinery and the resources within the mycelium, which mechanically increases the network complexity. The question that arises concerns the interest of relying on both branching types for the growth of the fungal network, so that they have been selected and kept.

*Podospora anserina*^14^ is a coprophilous filamentous ascomycete, a large group of saprotrophic fungi, that mostly grows on herbivorous animal dungs and plays an essential role within this complex biotope in decomposing and recycling nutrients from animal feces. *P. anserina* has long been used as an efficient laboratory model to study various biological phenomena, especially because it rapidly grows on standard culture medium, it accomplishes its complete life cycle in only one week, leading to the production of ascospores, and it is easily usable in molecular genetics, cellular biology and cytology.

As already discussed in^15^, the growth of hyphal network expansion and structure of *P. anserina* were characterized under controlled conditions. Temporal series of centimetric image size of the network dynamics, starting from germinating ascospores, were produced with a typical micrometric resolution. The image reconstruction steps were completely automated and allowed easy post-processing and quantitative analysis of the growth dynamics. By relying on the two main processes that drive the growth pattern of a fungal network, *i.e.* apical growth and hyphal branching, we have proposed^16^ a two-dimensional simulation based on a binary-tree model, allowing us to extract the main characteristics of a generic thallus growth. In particular, we showed that in a homogeneous environment, the fungal growth can be optimized for both exploration and exploitation of its surroundings with a specific angular distribution of apical branching. This numerical experience is obviously far from the *in vivo* growth of a saprophytic fungus such as *P. anserina*. However, it constitutes an excellent starting point to describe the thallus growth using mathematical concepts and language. Indeed, the objective of our mathematical modelling is to reduce a complex biological system into a simpler model, which is able to partly reproduce, or even better predict, the real system. A recurrent question is then to find the optimal degree of simplification for such a model, which should be neither too simple to avoid straying from realistic predictions, nor too complex to solve using numerical methods^17^.

The structure of this article is as follows. In the “Results” section, we first recall the methodology used to produce the model of the growing mycelium, then we describe the statistics extracted from observations that were incorporated into the simulation. These concern the lateral and apical branches, the curvature and the symmetry of the growth. Finally, we describe the density observable and derive predictions. In the “Discussion” section, we compare with direct observations made on the thallus growth in two configurations, the first on a standard culture medium, and the second on a low-nutrient medium.

## Results

### Modelling the thallus growth

Direct observation of the growth of the fungal network shows a monotonous, ever increasing complexity. This is particularly obvious via observables such as the number of branches, or the total length of the mycelium. This remains true, even when the culture medium is modified, with for example a quantity of nutrient depleted^16^. In the latter case, we found that the growth rate is affected but not the growth itself, *i.e.* only the value of the exponential growth parameter is affected. However, these observables are global aggregates and do not capture finer effects such as a change in the spatial distribution of matter. In this section we introduce a new observable, the density of the network, that combines both the amount of matter and its distribution.

In a previous article^16^, we discussed the foundations of a model built to describe the growth dynamics of the *P. anserina* branching network. In order to ease the reading of the work presented in the following, we recall here its main characteristics.

The observed network is composed of interconnected branches, called hyphae, whose ends are the apexes. Growth in hyphal length is achieved by adding material to the apex. The connections correspond to branches that may appear at the apex—called apical branching—or along a hypha—called lateral branching. We recall that for an apical branching, the *operating* hypha is the one which defines the widest angle with respect to the projection of the mother hypha, and the *exploratory* hypha is the branch defining the smallest angle^16^. The simulation of the network growth is based on the reproduction of these basic elements. The apical branching can only show one additional apex. We therefore rely on a binary tree, to which lateral branching events are allowed.

In the nomenclature used, $V_1$ are the tips (or apexes) of the branches, $V_3$ are the vertices corresponding to the connections between three branches, $V_ {1\ell }$ are the apexes of the lateral branches and $V_ {3\ell }$ are the nodes of these branches. In addition to these biologically defined objects, we can distinguish the crossings (overlaps) of hyphae from real vertices. These geometric intersections, called $V_ {3i}$ in the following, should not be confused with anastomosis (hyphae mergings). This distinction is necessary to compare with observations from the experimental conditions. During growth, the mycelium expands over a surface, but is not constrained in its upper part. Thus, overlaps can occur when two hyphae get close and cross each other, which makes the frequency of overlaps between hyphae important. In addition, the acquisition method, based on a light intensity contrast between the hyphae and the background, does not allow to discriminate an overlap from an anastomose event.

On the contrary, the simulation allows the distinction between $V_3$ and $V_{3l}$ from geometric vertices. This makes it possible to correct the magnitudes relating to the vertices $V_3$ and $V_{3l}$ that were observed experimentally. We check the relevance of the simulation by comparing the ratio of the vertices labeled 1-body ($V_1$ and $V_{1\ell }$) to the other vertices of the image, and this as a function of time, for both the experimental data and the simulation.

In the simulation, a set of random variables is used to fix the values of the growth of each apex and the branch angles. We give in^16^ the details of the probability laws used, as well as their parameters which were obtained by the analysis of the experimental data for some of them.

**Figure 1:**
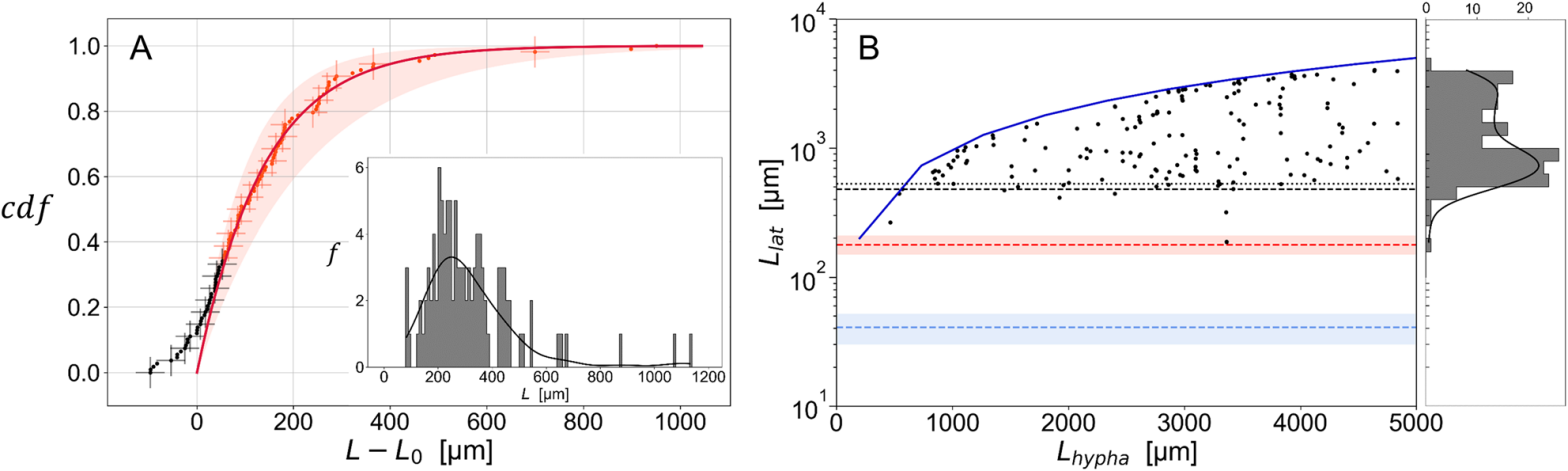
(**A**) Cumulative law of the distribution (in inset, bins 10 $$\upmu $$m) of $$N=109$$ lengths between two consecutive apical vertices $$V_{3}$$. The transition from black to red markers is defined by the maximum slope, found at $$230\pm 5\,\upmu $$m from the apex. The solid red line is an exponential fit of the data shown in red with $$1-2^{-\alpha (L-L_0)}$$. The data were manually shifted by $$L_0=180$$ $$\upmu $$m. Using a diagonal covariance matrix, the exponential fit parameters were found to $$\alpha =(10.4 \pm 3.9)\times 10^{-3} \upmu $$m$$^{-1} $$, $$R^2=0.99$$. We made use of R squared $$R^2=1-\frac{SS_r}{SS_t}$$, with $$SS_r$$ the residual sum of squares and $$SS_t$$ the total sum of squares to discuss the quality of the fit. The red area corresponds to one standard deviation. (**B**) Lengths $$L_{\text {lat}}$$ between the apex and a lateral vertex $$V_{3\ell }$$, measured when the branch appears in function of $$L_{\text {hypha}}$$, the length of the hypha when the branch appears. The dark blue solid line corresponds to $$L_{\text {lat}}=L_{\text {hypha}}$$. 95% (resp. 90%) of the data points are above the black dashed line at 480 $$\upmu $$m (resp. black dotted line at 530 $$\upmu $$m). The red dashed line corresponds to the apical dominance ($$L_0=180$$ $$\upmu $$m), as defined in A. The blue dashed line and area correspond to the mean and standard deviation of the distribution (not shown) of $$L_{\text {api}}=41\pm 11 \, \upmu $$m, defined as the length between the apex and the apical branch at the time of branching (see text for details).

#### Experimental observations implemented in simulation

In this section, we describe the observations implemented in the model following our previous work. In particular, we relied on statistical descriptions of the branches to discuss their spatial and temporal distribution.

##### Apical branching dynamics

In order to implement the apical branching statistics, we estimated the distribution of the distance *L* between two successive apical branches ($V_3$). The distribution and corresponding cumulative law are shown in Fig. 1A for experimental data.

Branching statistical behavior is separated into two distinct phases: a first phase of latency, also known as apical dominance and a second phase, which is well recovered by a memoryless law. The region of apical dominance, *i.e.* the length for which growth of the apex of the parent hypha dominates over the appearance of new apical branches, is estimated here to $L_0=180\,\pm 30 \upmu$m and the value of the rate of the exponential distribution to $\alpha = (10.4 \pm 3.9 )\times 10^{-3}\,\upmu$m$^{-1}$ (Fig. 1A,B). This phenomenon, which is well characterized in the literature (for a review, see^18,19^) but whose mechanisms are still poorly understood, implies that hypha extension is periodically predominant over the formation of new polarity axes in the vicinity of the apex. It should be noted that the apical branching is not located exactly at the apex but slightly behind it. This behaviour is named subapical branching in the literature^20,21^. In this work the expressions *apical branching* and *subapical branching* refer to the same process. The distance $L_{\text {api}}$ corresponding to the length between the branch and the apex was also measured. The corresponding mean and standard deviation of the distribution (not shown) were found to be $41\pm 11 \, \upmu$m and are shown with the blue line in Fig. 1B.

##### Lateral branching dynamics

The dynamics of lateral branching was found to be more subtle. First we discuss the correlation with the distance to the apex. We show in Fig. 1B the distribution of lengths $L_{\text {lat}}$ between a lateral branching and the corresponding apex, in function of the length of the hypha $L_{\text {hypha}}$. $L_{\text {hypha}}$ is the distance between the apex of the main hypha (on which the branching occurs) and a fixed point arbitrarily placed along this hypha. There is no apparent correlation between the lengths $L_{\text {lat}}$ and $L_{\text {hypha}}$. However, a region clearly emerges from the data, where the probability of observing a lateral branching is extremely low. We separated the population into two subparts, with one composed of 95% of the samples, as indicated by the black dashed line on Fig.  1B. We can therefore safely conclude that the lateral branches appear at a minimum distance of 480$\,\upmu$m from the apex. This length corresponds to the apical dominance behaviour observed for apical branching, but with an associated length about three times higher. Interestingly, the difference in apical dominance lengths is a clear parameter for distinction between the two types of branches. It is therefore trivial for an operator to distinguish between apical and lateral branches, providing that the temporal resolution of the collection of images of the network is sufficiently high. On the other hand, this distinction becomes more delicate with a single image available, particularly because the branching at the apex is subapical, as already discussed.

**Figure 2:**
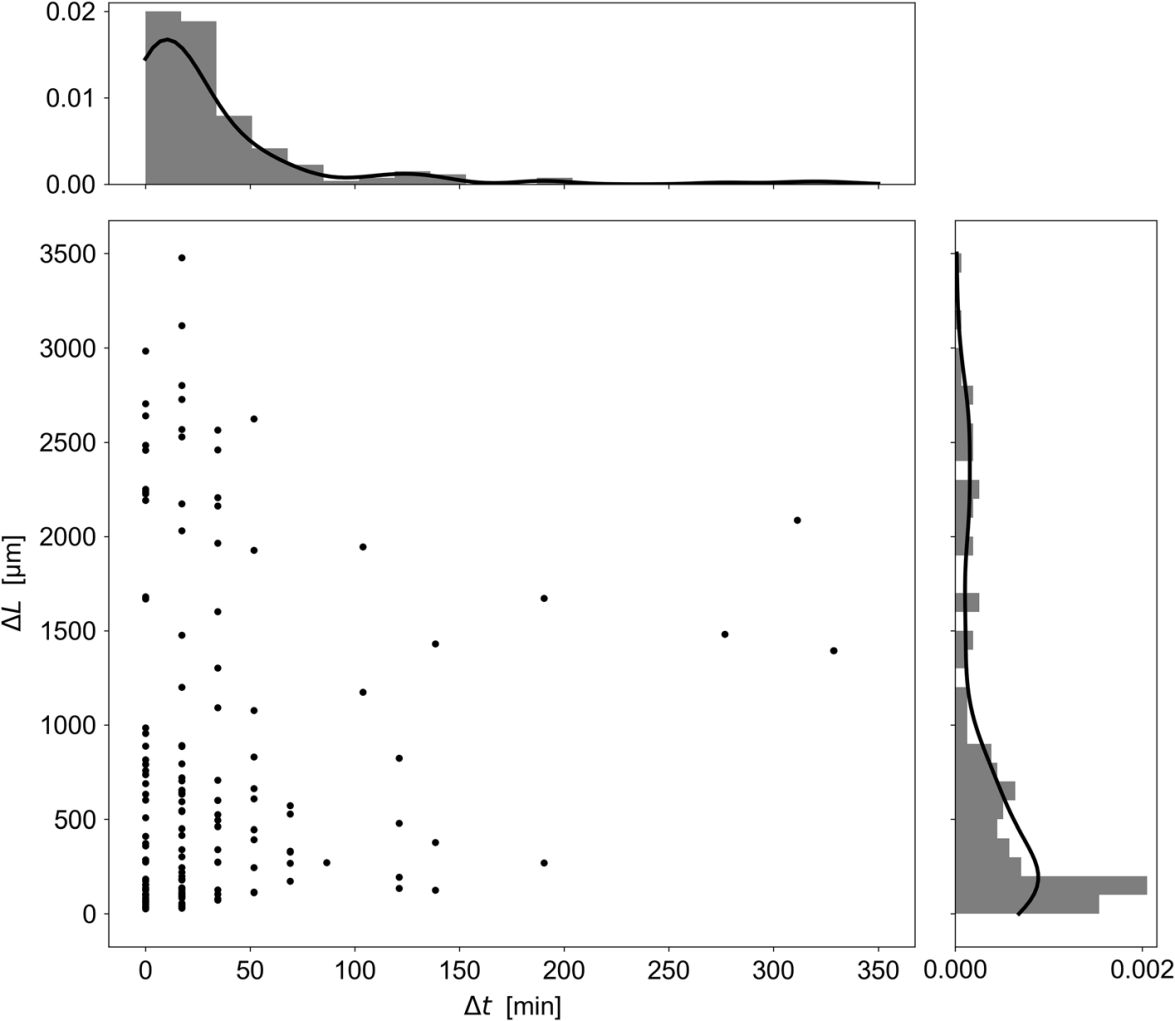
The main figure shows the spatial $\Delta L$ and temporal $\Delta t$ distance between two successive lateral branching, taken in chronological order, for $N=156$ branching events, from experiment on M2 culture medium (M2$_1$, see text for details). The solid black lines represent the kernel density estimate associated with the $\Delta L$ and $\Delta t$ distributions.

In the subapical region outside of the length of apical dominance, the appearance of lateral branches follow its own dynamic. We report in Fig. 2 the distance $\Delta L$ between two successive (in time) lateral branches, separated by a duration $\Delta t$. The temporal distribution can be adjusted using a memoryless exponential distribution (not shown), with rate parameter $(41\pm 19)\times 10^{-3}$ min$^{-1}$, $R^2=0.94$. Note *i*) that here and in the following, we have used the base-2 exponential function, unless otherwise stated ; and *ii*) that the laws are linearized to achieve the fit using the logarithm function. Here, $R^2$ is an indicator of the quality of the fit, the covariance matrix is assumed to be diagonal, as well as the logarithm of the law. In the spatial domain, we note the existence of two populations. The observed distribution is compatible with two decorrelated dynamics, that was fitted based on a combination of an exponential distribution and a continuous uniform distribution, by following the procedure described thereafter. We first fit the data using an exponential distribution $\Gamma _1 \, 2^{-\gamma _1\, \Delta L}$ and used the fit parameter $\gamma _1=(2.3\pm 0.3)\times 10^{-3}$ $\upmu$m$^{-1}$ ($R^2=0.80$) to proceed with a second fit, which is the mixture of the two distributions, *i.e.* $\Gamma _1 \, 2^{-\gamma _1\, \Delta L} + r$. We obtained $\Gamma _1\approx 28.2 \pm 2.3$ and $r \approx 1 \pm 0.5$ ($R^2=0.84$). By comparing the number of branches predicted by the uniform law on the considered length to the number of samples, *N*, we are then allowed to conclude that $22 \pm 11$% of the lateral branches population is driven by the continuous uniform distribution.

Therefore, lateral branching events seem to be driven by two simultaneous behaviors. On the one hand, the probability of branching in the vicinity of an existing side branch is greater than in other regions of the hypha. This probability is found to exponentially decrease with $\Delta L$. On the other hand, the probability of the emergence of a lateral hypha is uniformly distributed. Both populations are numerically of the same order of magnitude. Branches with a uniformly distributed probability distribution can trigger bursts of branches that are far away from the existing ones. These isolated branches give indications on which part of the thallus is growing. It is therefore interesting to determine their probability *p* per unit of length and time. As can be seen in the Fig. 2, this probability is not uniformly distributed over time. Most of the events take place for duration less than 90 min, indicating an aging effect. Thus, we propose an approximate value based on *r* and the duration of this activity window $p\approx (6 \pm 3)\times 10^{-3}\upmu$m$^{-1}$ 90 min$^{-1}$. In other words, the probability 1/2 to observe an isolated branch in less than 90 min is obtained for a length of approximately 80 $\upmu$m .

**Figure 3:**
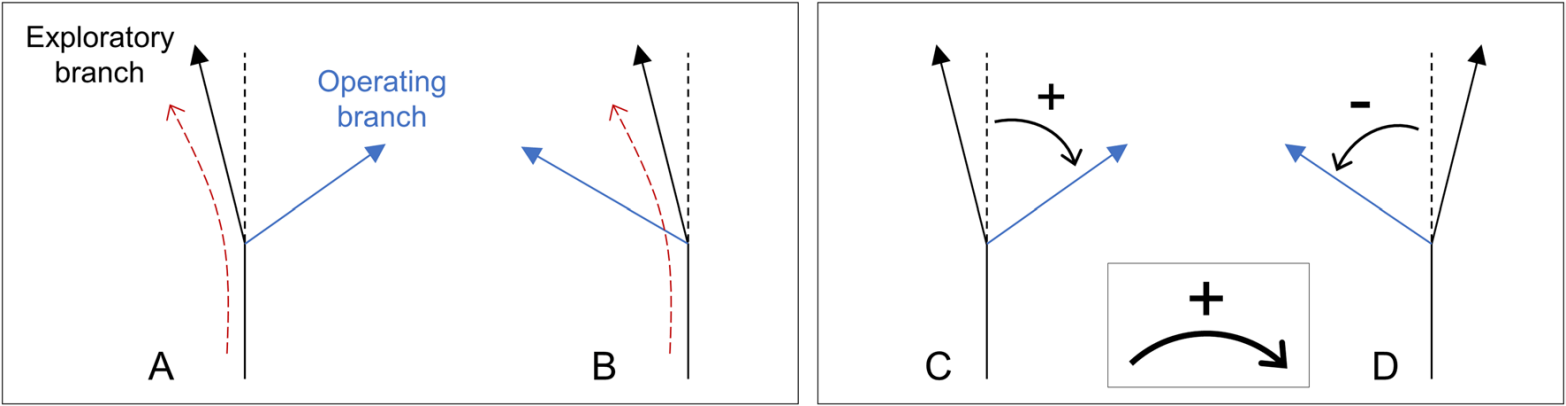
Symmetry of local (**A**,**B**) and global (**C**,**D**) branching. (**A**,**B**) Direction of subapical and lateral branching compared to the local curvature of the hypha (subapical branching is shown). A is in the opposite direction, B is in the same direction. We found 82% and 72% for subapical and lateral branchings respectively corresponding to configuration A. (**C**,**D**) Clockwise (**C**) and counterclockwise (**D**) direction of the subapical (large angle apical branching) and lateral branching. We found 54% and 56% for subapical and lateral branching respectively corresponding to configuration C. All collections are composed of 198 samples. The error is 5% in all configurations.

Finally we found that the scenario of lateral branching dynamics is (i) subject to a region of apical dominance and that (ii) two probability laws are needed to describe the distance between two successive branching events, highlighting two different mechanisms. Indeed, two successive branches can appear at a long distance from each other, suggesting a nucleation process depending on local random fluctuations of resources and cell wall building material^19,22,23^. The successive branches can also appear close to each other, which is the most likely configuration. This behavior may be the signature of an interaction with the environment: resources absorbed by an apex increase the concentration of cellular materials necessary to branch in the immediate vicinity of the apex. The branches then appear by burst in the vicinity of a first lateral hypha, that emerges without predictable location. In order to implement this complex behavior into the model, the nucleation of any lateral branches is determined by the local curvature of the branch during its growth, as discussed in the following and as described in Fig. 3A,B. Beyond a critical value, the position of the step is memorized and a probability law manages the emergence of a branch at each generation.

##### Spontaneous curvature

The observation of the trajectory of all the apexes clearly shows, apart from any branching process, that the growth is not rectilinear. In this paragraph, we give an estimate of the spontaneous curvature compared to the rectilinear trajectory, called tortuosity in the following. For that purpose, we define “a step of growth”, smaller than the distance between two apical branches. The orientation is determined by a probability law, parameterized according to the previous step. The probability law reads $A \, (\theta -\theta _0)^a \, (\theta +\theta _0)^b$ where $\theta _0$ defines the angular range, $a>0$ and $b>0$ are two constant shape parameters, and *A* scales the amplitude of the curvature. This is worth noticing that chirality is broken if $a \ne b$. Following the three types of branches described experimentally—the two branches originating from an apical branching (*exploratory* and *operating* branches^16^) and *lateral* branches, we made use of three sets of separate parameters for this propagation mode. Parameters were determined experimentally. It is interesting to note that while the spontaneous curvature of the hypha may be a marker of different spatial occupation strategies, it does not seem to impact the total length of the hypha produced by the network. The notion of tortuosity is quantified in the literature as the persistence length^24^, *i.e.* the measure of hyphae extension before presenting a change in direction. The latter is derived from the correlation between the angle formed at each step of the trajectory by the path followed and the tangent to this path. Each trajectory must be treated individually. For our application, we rely on a simple global measure. Therefore, we constructed a specific tortuosity as the normalized arc-chord ratio $\alpha = (L_{\text {tot}} - L_p) /(L_{\text {tot}}+L_p)$ of the length $L_p$ of the network composed only of nodes of degree 1 and 3, *i.e.* pruned from the curvature, with the total length of the network $L_{\text {tot}}$. Tortuosity was found in accordance for standard culture medium M2 and low-nutrient medium M0 (resp. $\alpha =0.038\pm 0.005$ and 0.041$\pm 0.005$), without any particular correlation in time or space. Although this is not the case in this work on M2 and M0 media, $\alpha$ can be expected to be different in the case of mutant strains showing less rectilinear elongation. In agreement with the low values of $\alpha$ found in this work, we implemented this notion in the simulation as follows. On average at each time step, the collection of cross products of two successive velocity vectors of the same apex is zero.

##### Branching chirality

We verified that apical and lateral branches do not spontaneously generate a global symmetry breaking (*i.e.* chirality breaking) by comparing a collection of branches with a defined positive clockwise rotation direction, as shown on Fig. 3C,D. To this end, we have built four collections corresponding to the two culture media and the two types of branching. They are composed of 198 samples each. For apical branches on M2 growth medium, which we define as the reference condition, a binomial test is used to assess whether the frequency of occurrence of positive orientations (measured at 54%) deviates from a theoretical probability of 0.5. The observed *p*-value was estimated at $\approx 0.27$. Orientation in the positive or negative direction is then considered as equiprobable. We then compared the apical and lateral branches on culture media M2 (56% for lateral hyphae) and M0 (56% and 58% for apical and lateral hyphae respectively) to the reference using binomial tests adjusted for multiple comparisons using Bonferroni correction (control vs. all treated groups, like in Dunnett’s procedure). The *p*-values were found to be well above 0.05. We can then conclude that probabilities of clockwise and counterclockwise orientation are all found to be consistent with the equiprobability assumption.

The subapical rather than apical nature of the apical branching was described previously. Combined with the spontaneous curvature of the hypha (see the paragraph *Spontaneous curvature*), one can wonder whether the orientation of the operating hypha depends on the local curvature (whether branching can be observed inside out or outside out with respect to the curvature), as can be seen in Fig. 3A,B. We have reproduced the previous procedure to test this hypothesis. For the binomial test with a probability of success $p=0.5$ applied to the reference condition, we found a *p*-value much lower than 0.05, allowing us to clearly reject the equiprobability hypothesis: during an apical branching, the exploratory and operating hyphae are probably located on both sides of the mother hypha, as shown in Fig. 3A. Surprisingly, the *p*-value adjusted to the reference is found to be $2.10^{-7}$ on the low-nutrient culture medium (M0), which rules out the correlation with the orientation of the mother hypha. Finally, we can retain that the right/left position relative to the parent segment of a lateral and apical operating branch is only given by the curvature of the parent segment, except in the case of the M0 culture medium, for which it is not possible to measure any correlation.

**Figure 4:**
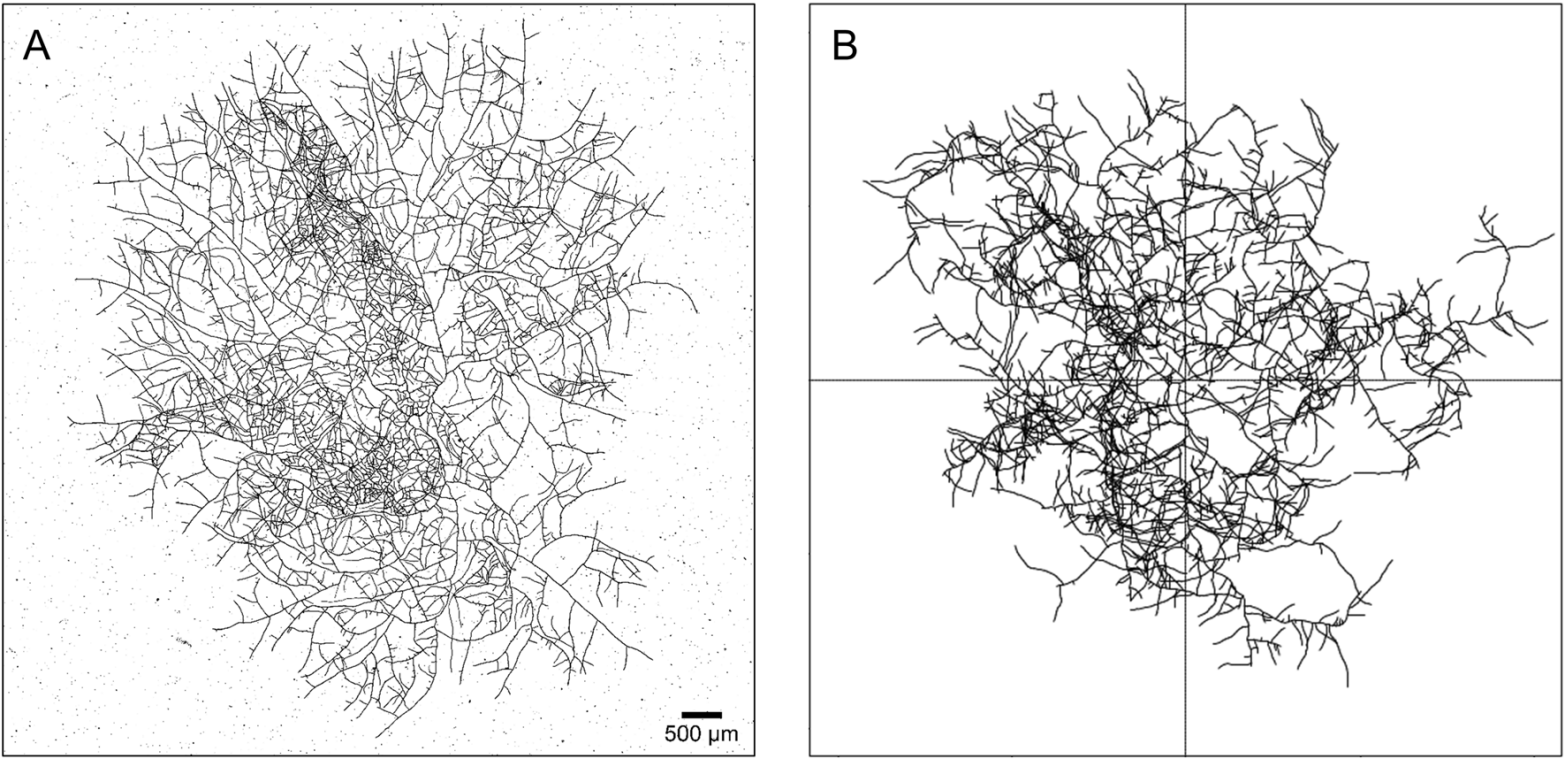
(**A**) Thallus of *P. anserina* reconstructed from $3 \times 4$ tiles, extracted from experiment previously discussed in^15^ at $t=15$ h after the ascospore germination. (**B**) Simulation of the growth of *P. anserina* after the same duration of growth, the simulation time being scaled on experimental time.

All the observations described in this section were used to feed the simulation. This more complete and realistic description allows us to take into account some of the more subtle effects observed, while introducing a relatively small number of empirical parameters as well as probability laws. As for the previous simulation, time and space parameters were calibrated from the experimental data. Typical experimental data and simulation after 15 h of growth are compared in Fig. 4.

#### Different growth phases

**Figure 5:**
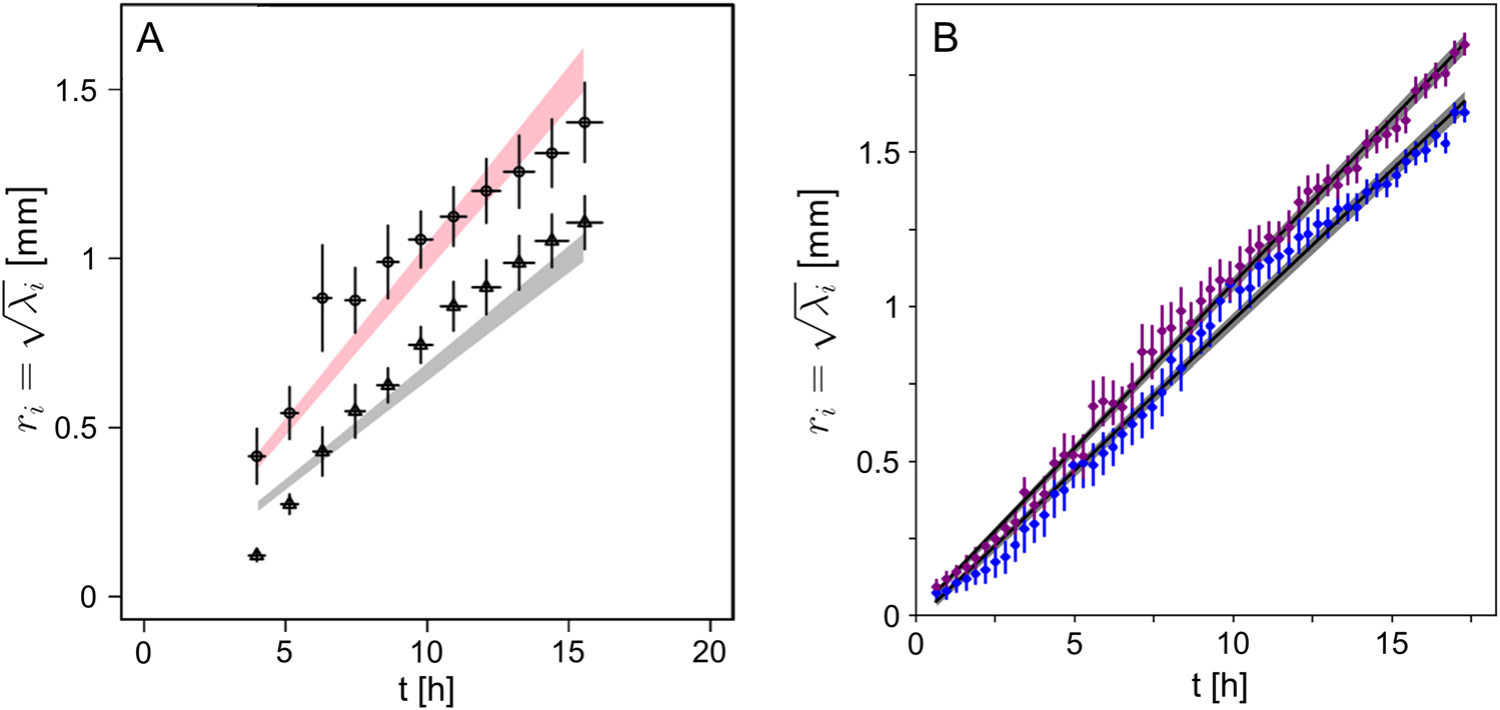
Roots of the eigenvalues $r_1 = \sqrt{\lambda _1}$ and $r_2 = \sqrt{\lambda _2}$ of the apexes ($V_1$) cloud distribution—see Eq. (1)—as a function of growth time. (**A**) Corresponds to the modelling of the growth, points correspond to the output data of simulation, shaded area are the theoretical function $r_i = B_i \, t$. (**B**) Corresponds to experimental data, obtained from experiment M2$_1$, see text for details. The grey area shows one standard deviation for both fits. Corresponding slopes $B_i$ are $0.098 \pm 0.002$, $0.107 \pm 0.002$ mm h$^{-1}$ with $R^2>0.99$. In this last case the data have been manually shifted by $t_0=1~$h±10 min. Corresponding sphericity $2\,\lambda _2 / (\lambda _1 + \lambda _2)$, with $\lambda _1 \ge \lambda _2$, is found to be constant at 0.88$\pm 0.02$.

The monitoring of the growth of the network can be quantified at each moment thanks to the counting of the vertices of different natures in the network. This work is made simple in the case of the simulation because there is no ambiguity about the qualification of the different nodes: $V_1$, $V_{1\ell }$, $V_3$, $V_{3\ell }$. In the following, we will rely on the number of $V_1+V_{1\ell }$ (apexes) vertices as a function of time to monitor the growth of the thallus. However, these quantities do not contain any information on the spatial distribution of the apexes and are not sufficient to regain the different phases of growth. Building on the counting of $V_1+V_{1\ell }$ , we define the *density* as a new characteristic observable that we write $\rho _o(t)=\frac{N_{1}(t)}{S_{1}(t)}$ where $N_{1}(t)$ is the number of all 1-body vertices ($N_{V_1}$ and $N_{V_{1\ell }}$) and $S_{1}(t)$ is a characteristic surface generated by the spatial distribution of $V_1$. In the case of a filamentous network, the notion of surface is tricky. As a first approach, we can rely on the surface occupied by the fraction of the substrate covered by hyphae. The latter can be simply calculated using the total length but does not contain information on the sprawl of the network. Another approach is based on the surface defined by the outer ring, using *e.g.* the convex hull. In this case, the density (*i.e.* hyphae length per unit surface) of the network in the surface is not taken into account. We rely in this work on the distribution of apex locations, in particular to discuss the competition between lateral branches favoring densification and apical branches favoring exploration. We propose to define this surface as the square of the characteristic length generated by the spatial distribution of the $V_1$ vertices. In a previous work^16^, we introduced the inertial tensor *I* of the spatial distribution of the vertices $V_1$. We will briefly recall in the following the derivation method of the characteristic length. Let us first write the expression of this tensor :

1 $$\begin{aligned} I&= \begin{vmatrix} \sum _n (x_n -x_0) \, (x_n -x_0)&\sum _n (x_n -x_0) \, (y_n -y_0) \\ \sum _n (x_n -x_0) \, (y_n -y_0)&\sum _n (y_n -y_0) \, (y_n -y_0) \end{vmatrix} \end{aligned}$$

with $(x_n,y_n)$ the coordinates of the $V_1$ (apical) collection of locations at time *t* and $(x_0, y_0)$ the average of this collection. Diagonalization of this tensor allows us to derive two eigenvalues ($\lambda _1$ and $\lambda _2$) and two eigenvectors, which are the main axes of the $V_1$ vertices cloud. These eigenvalues correspond to the dispersion of the vertices in the plane and have the dimension of the square of a length. We can then directly derive a surface by calculating the square root of their product $\sqrt{\lambda _1 \, \lambda _2}$. This surface is characteristic of the $V_1$ distribution for each time step and we propose to use it as a proxy for $S_{1}$. We show in Fig. 5A the temporal variation of the two roots of the eigenvalues, $\sqrt{\lambda _1}$ and $\sqrt{\lambda _2}$, of the $V_1$ vertices extracted from networks obtained using both a simulation and an experiment.

The spatial extension of the thallus is constrained by its boundary conditions. The surface must be zero for $t=0$ and must converge asymptotically to a finite value in the long run. These constraints must be reflected in the chosen law to adjust the time behaviour of the eigenvalues. The law which best describes the evolution of the roots of eigenvalues over time is $r_i(t)=\sqrt{\lambda _i(t)}=A_i \, (1-2^{-a_i \, t})$ where *i* refers to the two eigenvalues $\lambda _1$ and $\lambda _2$, and $a_i$, $A_i$ are positive constants. Apart from the spatial extension, both longitudinal and transverse growth velocities of the $V_1$ distribution can be extracted by deriving $r_i$ with respect to time, *i.e.* $v_i(t)=\partial _t r_i(t) = A_i \, a_i \, \log _e(2) \, 2^{-a_i \, t}$.

We can therefore rewrite the density in a more convenient form, as the ratio $\rho (t)=\frac{N_{V1}(t)}{r_1(t) \, r_2(t)}$. For the growth period considered in this work, $r_i(t)$ can be safely approximated using a linear function $r_i=B_i \, t$, as can be seen in Fig. 5. In^16^ we have shown that $N_{V1}$ can be written as $N_{V1}(t)= C\,2^{\omega \, t}$, with $C>0$ and $\omega >0$, and we estimated these last parameters. We can therefore derive the following expression for the density, $\rho (t) = D\,\frac{2^{\omega \, t}}{t^2}$, with $D=\frac{C}{B_1\,B_2}$. The density diverges for both $t\rightarrow 0$ and $t\rightarrow \infty$. In other words, density will show a minimum for an intermediate time, $t_{\text {min}}$, which defines two distinct growth phases. Note that a minimum always exists if $S_{1} = P_n(t)$, with $P_n$ a polynomial of order *n* (with the term $n=0$ to respect the initial condition $S_{1}(0)=0$) or if the time dependence characteristic length generated by the $V_1$ cloud is of Brownian type (*i.e.* $\sigma \sim \sqrt{t}$). Basically, if the characteristic $S_{1}$ area is a *t*-polynomial, two distinct growth phases are identifiable on either side of $t\simeq 1/\omega$, where $\omega$ is the characteristic growth parameter of the number of $V_1$. We can then derive an estimation of $t_{\text {min}} = \frac{n}{\omega \,\log _e\,2}$, with $n=2$ if the eigenvalues grow linearly in time.

**Figure 6:**
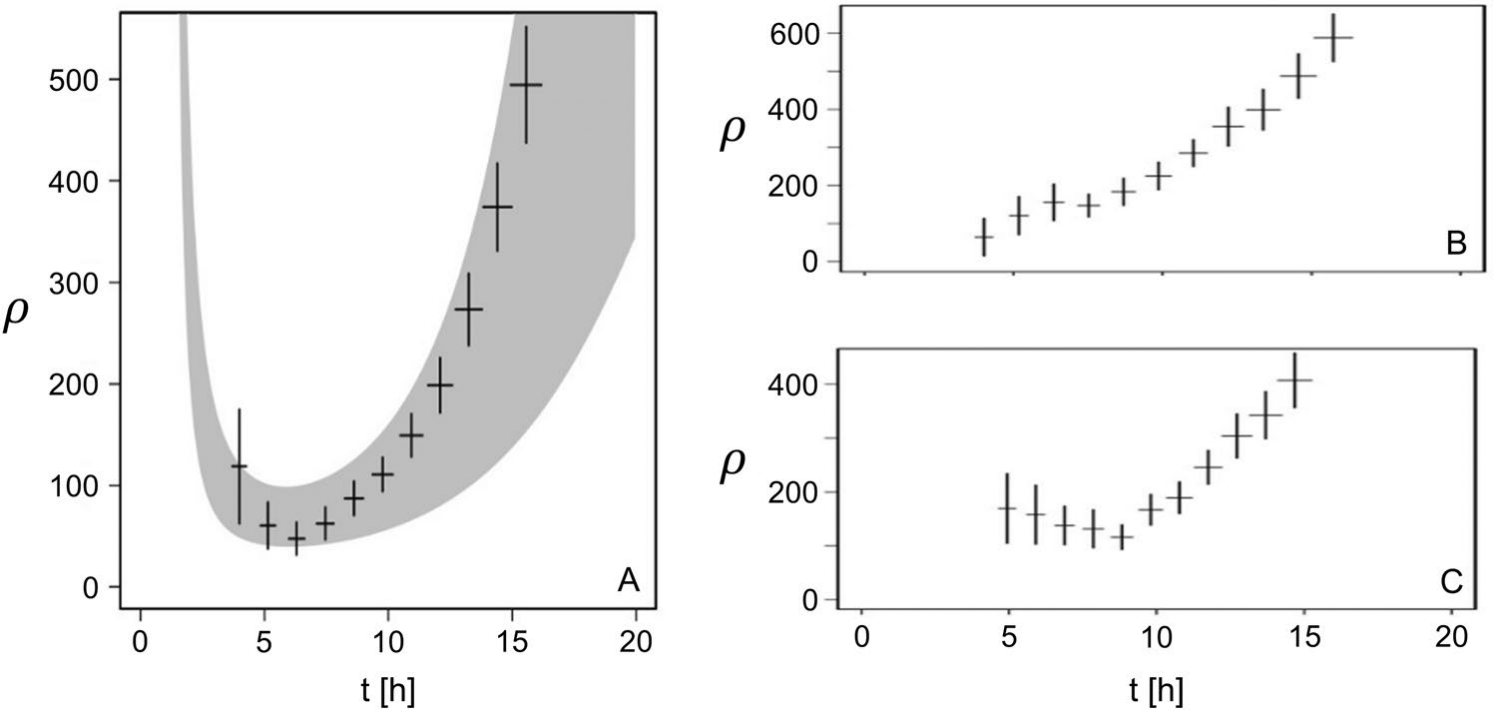
(**A**) Density versus time for output data of simulation (points) and theoretical function $\rho (t) = D\frac{2^{\omega \, t}}{t^2}$ with adjusted parameters (grey area for one standard deviation). (**B**) same as (**A**) but with many lateral branches and big angle for operating branch. (**C**) same as (**A**) but without lateral branches and small angle for exploratory branch.

Let us now compare the density $\rho _o(t)$ obtained from the simulation with the theoretical form of $\rho (t)$ discussed previously. First, we adjusted the spectra of $N_{V1}(t)$, $r_1(t)$ and $r_2(t)$ independently in order to derive *D* and $\omega$, allowing to regain $\rho (t)$. The corresponding density is shown in Fig. 6A. The grey area corresponds to the theoretical form of $\rho (t)$ with one standard deviation (see below). The points correspond to the $\rho _o(t)$ densities measured individually at each time step from the simulation. The uncertainties for the $\rho _o(t)$ points and for the parameters introduced in the theoretical form of $\rho (t)$ are calculated using the bootstrap method and relying on the Poisson hypothesis for counting process. The general behavior with a marked minimum is regained in both cases. The minimum of the simulation output (corresponding to $\rho _o(t)$) is found approximately for $t_0^{min}\simeq 6$ h, compatible with the value of $t_{\text {min}}$ calculated by assuming a linear behaviour of the eigenvalues, $t_{\text {min}} \simeq 5$ h (corresponding to $\rho (t)$).

We show in Fig. 6B,C respectively, alternate results from the same analysis method, but with additional constraints. First, we have chosen parameters that maximize the density at the centre of the network, by increasing the lateral branching frequency and setting the angle of the operating branches to a large value, *i.e.* 120°. The production of new material is then favourably located in the vicinity of the centre (Fig. 6B). Second, we maximized the number of $V_1$ vertices near the outer ring by prohibiting lateral branching and by setting the angle of the operating branches to the small value of 10° (Fig. 6C). All material production is then concentrated at the tips of the apexes located favourably in the outer crown. Since the density is written as the ratio of an exponential to a polynomial, we found in both cases that the density grows in the long run. With the chosen parameters, the branching frequency is higher than the reference in the first case and lower in the second. The minimum density being expected for a growth time $t_{\text {min}}$ which varies in $1/\omega$, we regain that the value of $t_{\text {min}}$ obtained for the reference is framed by the values obtained for the two proposed variants. We can then distinguish two specific functions for the apical and lateral branches. The former are related to the occupation of the long distance regions defining a perimeter, in which the latter will locally densify in order to exploit the available nutrients.

To conclude, we propose the following predictions regarding the mycelium growth. Following germination in a homogeneous environment, the growth of the network presents three distinct phases, which can be observed through the density.

- During the first growth phase, which lasts approximately 6 h, the growth dynamic maximises the space explored, in order to optimize the colonisation of its distant environment and to favour the future exploitation of available resources. During this extension phase, the density is mechanically reduced.
- In the second phase, the lateral branching process appears and balances the mass distribution in the network, *i.e.* the density remains stable over a period comparable in duration to the first growth phase.
- During the third phase, exploitation of the colonized area, *i.e.* the capture of resources, becomes the dominant behavior, thereby inducing a significant increase in the density. To this end, the mycelium of *P. anserina* produced lateral branches from any point in its network, which fixes it permanently in its environment. The development of the occupation of the already explored surfaces allows (i) to provide resources for the whole network, especially for regions that are far from the apexes and (ii) to avoid the presence of other competing organisms.

Finally, it is expected that the time at which the density reaches its minimum $t_{\text {min}}$ depends only on the growth rate $\omega$ of the thallus. These different growth periods are not specific to the *P. anserina* fungal network, whose growth can be modeled as a binary tree. For example, different metabolic phases in human life course were reported^25^. In this case, the marker used is the daily energy expenditure, or in intensive form, the energy mass density.

### Experimental quantification of the thallus growth

In previous works^15,16^, we described the acquisition process and data processing of images of the *P. anserina* whole thallus. We obtained from the growth of the mycelium a collection of images regularly spaced in time (about 18 min) during the first 20 h of growth. The spatial resolution of the images (about 1 $\upmu$m) allows the observation of the fine structure of the mycelium, consisting of hyphae of 5 $\upmu$m in diameter. A graph of the network formed by the thallus is then reconstructed from each image of the collection (see Fig. 4). In this graph, the tips (apexes) are degree 1 vertices, and their number is noted *A*. The network itself is composed of hyphae (degree 2 vertices), whose total length is noted *L*. We used these generic data to calibrate the experimental simulations, as described in “Modelling the thallus growth”.

**Figure 7:**
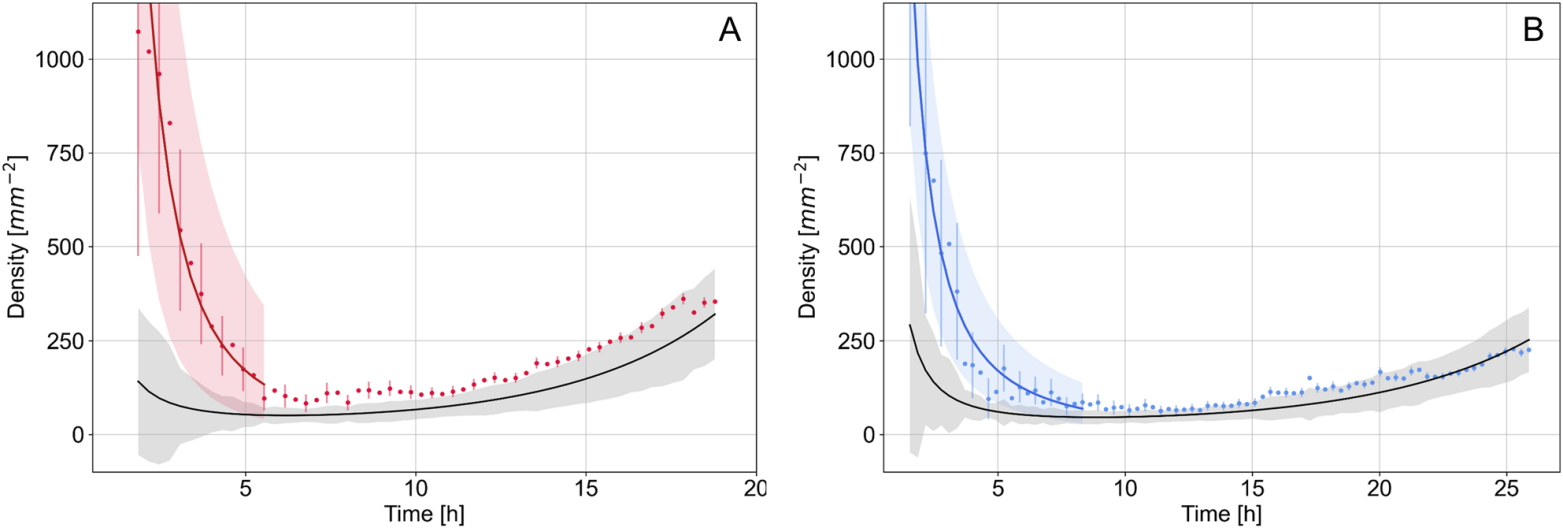
Experimental density in function of time. Culture media are respectively M2$_1$ (**A**) and M0$_1$ (**B**). Points are density computed at each time step, black solid line is density $\rho$ computed from expected laws of apexes number *A* and surface *S* growth. Grey area shows one standard deviation error. Solid red and blue lines are fit based on $\beta \, t^{-\alpha }$. Red and blue areas show one standard deviation. Respectively for M2 and M0 culture media, $\alpha$ were found to 2.1$\pm 0.3$ and 1.6$\pm 0.3$ with $R^2=0.96$ and 0.95. With $\alpha =2$ (not shown) we found $R^2=0.96$ and 0.76.

In the following, based on the time series *A* and *L* obtained during the growth of the thallus from an ascospore and over a period of typically 20 h in a controlled environment, we propose to discuss the temporal dynamics of the density. According to the definition proposed in the previous “Modelling the thallus growth”, we construct the density at each time step using the eigenvalues of the collection of the tips locations and the number of apexes *A*.

*Standard culture medium M2* The starting point of each experiment is a germinated ascospore placed in standard growing conditions, at a temperature of 27$^\circ$C. We carried out three independent experiments to obtain complete series of images of the thallus growth under two different conditions: growth on M2 culture medium, or M0 culture medium. The first medium (M2) is the most commonly used for *P. anserina* growth and reproduction in vitro. The carbon source is dextrin, a polysaccharide derived from starch. The M0 culture medium composition is the same as M2, but without any carbon source. However, it should be noted here that *P. anserina* is able to partially degrade the cellophane used to maintain the fungus in two dimensions^14,15^. However, this carbon source is largely in the minority compared to the standard M2 culture medium. All the protocols including standard culture conditions and media composition for this microorganism can be accessed online (see^14,26^). Experiments are named respectively M0$_i$ and M2$_i$, with $i=1,2$ or 3 thereafter.

The general base 2 exponential form for a limited growth time of the number of apexes $A= C\,2^{ \omega \,t}$ has already been extensively discussed in^15,16^, especially the extraction of the parameters $\omega$ and *C*. For M2 medium, the doubling frequency is found in this work respectively at $0.48\pm 0.03$, $0.46\pm 0.02$, $0.49\pm 0.03$ h$^{-1}$ (see Table 1).

We will focus in this work on the evolution of the surface, defined here as the product of the eigenvalues of the cloud formed by the collection of the apexes locations at each time step. We have represented in Fig. 5B the temporal evolution of $$ r_1$$ and $$ r_2$$, for the M2$$_1$$ experiment. Both of them clearly follow a linear law, whose slopes $$B_1$$ and $$B_2$$, about 100 $$\upmu $$m.h$$^{-1}$$, are found to be comparable between them, as well as compatible with the evolution predicted during the simulation.

This allows us to derive the temporal evolution of the density. We have represented density in Fig. 7A for the experiment M2$$_1$$. We regain the three-steps evolution of the density, showing a pronounced minimum. We have also represented $$\rho $$ in superimposition for the same experiment, computed from expected laws of the number of apexes *A* and surface *S* growth. The behavior for the smallest times is found in the trend but the dynamics is clearly lower. The curves are in very good agreement as soon as the growth time exceeds approximately 7 h, in the vicinity of the density minimum. In order to allow the comparison with the simulation, we extracted the growth rate $$\omega $$ and the corresponding prefactor *D*. In accordance with the simulation, we found 0.47$$\pm 0.03$$ h$$^{-1}$$ for $$ \omega _i$$ and $$222\pm 50$$ h$$^2$$ mm$$^{-2}$$ as average value of $$ D_i$$ for the three experiments on M2 culture medium. We can also derive an estimation of $$ t_{\text {min}}$$ using $$t_{\text {min}} = \frac{n}{\omega \,\log _e\,2}\approx 6.0\pm 0.3$$ h, when the eigenvalues are both linear in time ($$n=2$$). The values extracted from the different experiments and for the simulation are summarized in the Table 1.

**Table 1:** Summary of numerical values for the data from simulation and from the experiments, allowing to regain the density. $\rho (t) = D \, \frac{2^{\omega \, t}}{t^2}.$

	Simulation	Experiments					
		M2$_1$	M2$_2$	M2$_3$	M0$_1$	M0$_2$	M0$_3$
$\omega$ [h$^{-1}$] $\times 100$	59 ± 0.5	48 ± 3	46 ± 2	49 ± 3	37 ± 2	33 ± 2	33 ± 2
*D* [h$^2$.mm$^{-2}$]	454 ± 210	194 ± 44	266 ± 54	207 ± 42	416 ± 89	489 ± 94	420 ± 80

$\omega$ is the exponential argument of the number of apexes and $D=\frac{C}{B_1 B_2}$ is obtained from the combination of the initial number of apexes *C* and the results of the eigenvalues fitting procedure (see Fig. 5 and text for details). To facilitate reading, $\omega$ values were multiplied by 100.

*Low-nutrient culture medium M0* The partial agreement observed in the initial period of growth is based on growth on M2 culture medium. The question that arises is to discriminate whether this initial dynamic originates from an effect of the external environment, *i.e.* the available resources or a growth stress, or if this behavior is driven by cellular processes inscribed in the genotype of the fungus. In the latter case, the initial growth dynamic is not expected to be affected by the availability of resources or a stress. How do the phases evolve when the metabolism is slowed down by a nutrient-depleted environment? For this purpose, we conducted a second triplicate experiments with a low-nutrient medium M0, called M0$_i$, with $i=1,2,3$ in the following.

As expected^16^, both slopes of the eigenvalues $B_1$ and $B_2$ and the growth rate $\omega$ are reduced by one fourth, respectively to approximately 75 $\upmu$m h$^{-1}$ and 0.34$\pm 0.02$ h$^{-1}$ in a nutrient-depleted environment. Consequently, $D\approx 450\pm 50$ h$^2$.mm$^{-2}$ is found to be twice higher than in the M2 condition (see Table 1). A one-way analysis of variance on the two experimental conditions indicates a significant difference between M2 and M0 means ($222 \pm 38$ vs. $442 \pm 41$, $F(1,4)=45.72$, $p=0.0025$), while a robust test for the equality of variance (Levene’s test) suggests biological variances can be considered equal across samples ($p=0.8411$).

However, the general dynamics are preserved, leading to density dynamics equivalent to the behavior reported previously, with a good agreement beyond the first growth phase and a very marked minimum around 10 h.

## Discussion

The development of numerical imaging accompanied by the automatization of acquisition processes and image processing has recently opened a new experimental period with the development of numerous devices, whose objective is the extraction of dynamic quantities characteristic of the network architecture from images of a growing mycelium. These experimental devices have become increasingly efficient in extracting statistical data, without the need for a precise understanding of the molecular and cellular mechanisms governing hyphal growth or branching processes in particular^19^. Studies can be conducted at the hypha scale^24,27^, or at the mycelium scale in two dimensions^28,29^, or three dimensions^30,31^. In addition, quantitative observation of mycelium can support the development of fungal network modeling (as in^30^). In this context, based on a simulation of the *P. anserina.* mycelial network as a binary tree, and calibrated from experimental data, we seek to understand how the mycelium optimizes its expansion and densification. Thus, in a previous work, we showed that the observed distribution of apical branching angles corresponds to the maximization of radial extension of the thallus, while minimizing overlaps^16^. The study conducted here, has allowed us to propose advantages related to each of the two types of branching, *i.e.* apical and lateral branching. For this purpose, we were interested in the density of apexes, studied within the framework of the network simulation on the one hand, and with experimental observations on the other. The density observable has the great interest to take into account both the quantity and the spatial distribution of the hyphal material.

**Figure 8:**
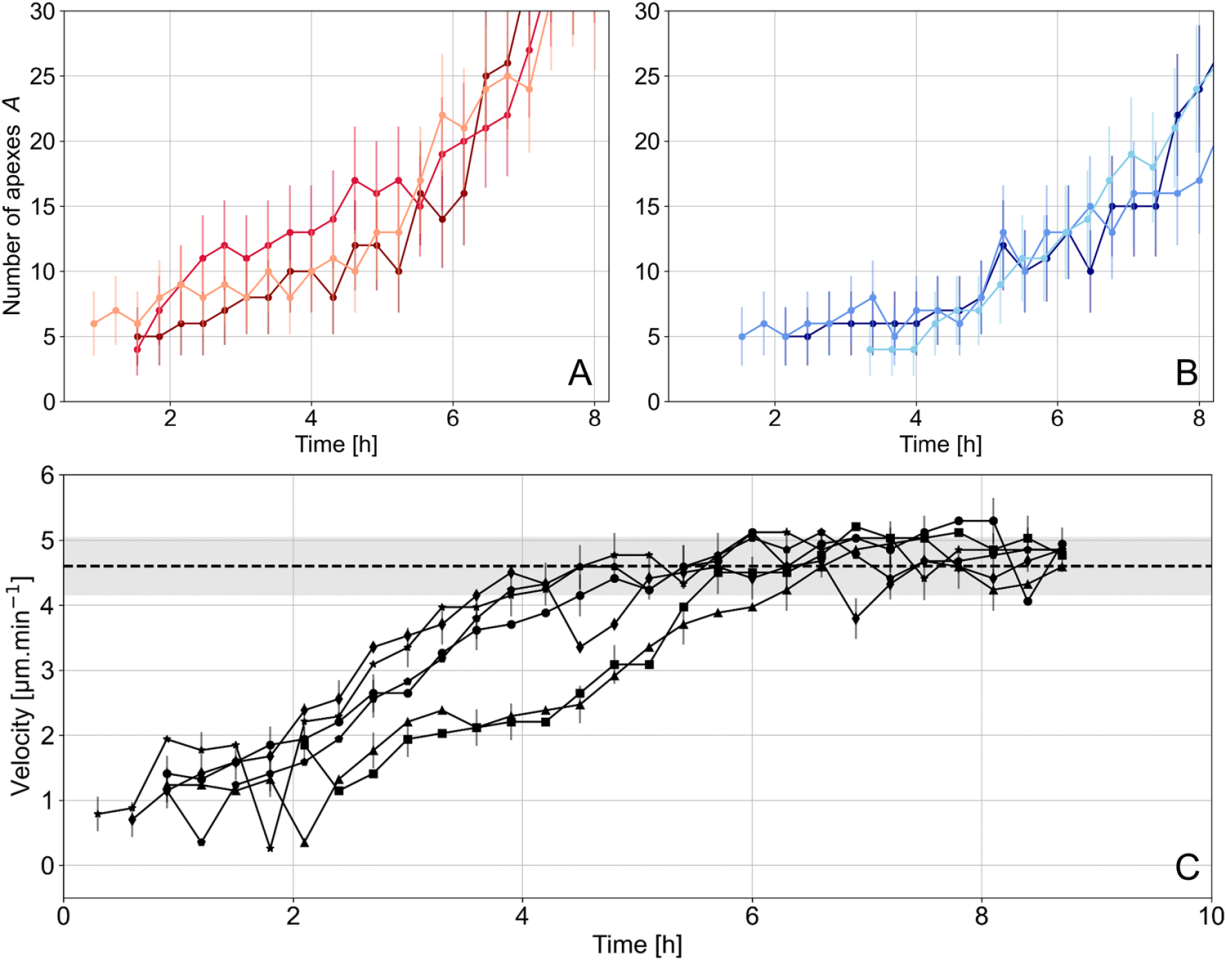
Number of apexes as a function of time for the three replicates on M2 medium (**A**) and M0 medium (**B**) respectively. Only the initial period of growth is shown, defined up to the average time $t_{\text {min}}$ for the three replicats on M0 medium. (**C**) Respective velocities of the six initial hyphae. Dashed line corresponds to the average of the asymptotic velocities of 20 apical hyphae (not shown). The grey banner represents one standard deviation of the distribution of these values.

The various extractions made on the dynamic behavior of the mycelium in “Modelling the thallus growth” allowed us to feed the modeling of the thallus growth to derive fine predictions. Thus, the density $\rho =\frac{A}{r_1\,r_2}$, constructed as the ratio of the number of apexes *A* and the product of the roots of the eigenvalues ($r_1$, $r_2$) of the inertia matrix *I*, consisting of the collection of the apexes locations. The expected behavior is composed of three phases of growth. The first one is a spatial extension, characterized by a rapid decrease of the density. The second is a phase of homogeneous extension, where extension and exploitation grow in a density-conserving manner. The third phase shows a dynamic of intense exploitation, where the density increases. A density minimum is found, which depends only on the growth rate $\omega$ of the number of apexes *A*. These phases seem generic and are also observed in human metabolism for example^25^.

We can then compare with direct observations of the growth of *P. anserina*. In both cases, the typical density dynamics is found, especially after a long time of growth, with a marked minimum, showing a transition between a first phase of decrease and an other one of density growth. A deviation with the initial phase of density decay is also found. The times at which the density reaches its minimum on the standard M2 medium, used to calibrate the model, and in the simulation are compatible. They are found around 6 h in both cases. It is interesting to note that this time is a function of only the polynomial describing the increase of the occupied surface through time, whose order is fixed in this work at $n=2$, leaving as the only adjustable parameter the growth rate of apexes number $\omega$, *i.e.* $t_{\text {min}}\propto 1/\omega$. Growth on a low-nutrient medium M0, for which $\omega$ is lower by about a quarter compared to the M2 case leads to a higher $t_{\text {min}}$, found here of about 10 h. The phases of densification are then independent of the available resource, which will only have an impact on the speed at which the different phases are explored.

We are now interested in the initial difference between the observed and expected density. Recall that the density is calculated as the ratio of the number of tips *A* and the product of the roots of the eigenvalues of the collection of apexes locations $$ r_1$$, $$ r_2$$, which is expected to vary as $$t^{-2}$$. At long growth time, the behavior of *A* is exponential: $$A\propto 2^{\omega \,t}$$. On the contrary, at short time, when the number of apexes is small, *i.e.* close to unity, the growth does not correspond anymore to the proposed law, and is rather linear (see Fig. 8A,B).

Two processes are responsible for the growth of the number of apexes *A*: apical and lateral branches. The former are specialized in the exploration of the environment^16^, while the latter are related to the densification of the network. Theses are crucial in the growth of the apexes number. The branching statistics follow a random distribution for both types of branching, preceded by a forbidden region, also called apical dominance (see Fig. 1). However, the numerical values associated with these laws are not identical. The region of apical dominance is of the order of 230 $\upmu$m, while that of the lateral branches is found to be of the order of 500 $\upmu$m. This difference sets the two growth phases. The first lateral hyphae appear when the initial apical branches exceed the critical length. In the first hours of growth, the velocity $v_h$ of the hyphae from the ascospore was found to grow steadily from 0 to 4.5 $\upmu$m min$^{-1}$ (see Fig. 8C). Therefore, a new densification phase should appear after about a duration $\Delta t$ such that $\int _0^{\Delta t} v_h ~ \text {d}t = 500$ $\upmu$m, or 7 h of growth.

The question that arises then is to identify whether the initial growth dynamics depends on the available resources, or if it is controlled by the initial conditions (as it can be observed for seeds), *i.e.* the stock of resources present in the ascospore, assumed to be identical in all the experiments carried out. Based on the reasonable assumption of *i*) a linear dependency of the roots of the eigenvalues of *I*, *i.e.* $r_1 \, r_2 \propto t^{-2}$, and *ii*) *A* is constant in the initial period, then we must find that the density varies as $t^{-2}$. We show in Fig. 7 the fit of the experimental data with a $\beta t^{-\alpha }$ law in the time range $[0-0.9 \, t_{\text {min}}]$, with $t_{\text {min}}$ the time such that the minimum of the density is $\rho (t_{\text {min}})$. It is clear that the proposed fit is much better than the law derived from the long time behavior.

Now let us discuss the numerical values of the $\alpha$ exponents found, in relation with the proposed behavioral law. The exponents found are respectively 2.1, 2.5 and 2.3 for M2 and 1.6, 1.9, 1.1 for M0 culture medium, all uncertainties being smaller or equal to 0.3. These exponents are clearly higher in the M2 case, indicating a higher growth rate of *A* the apexes number, but remain in all cases compatible with a slope in $t^{-2}$.

Finally, we can test the hypothesis of independence of the initial growth dynamics from the available resources. For this, we set $\alpha =2$, and then check the dependency of the fit on the boundary conditions. We found that $R^2$ was in the range 0.6 to 0.9 when the exponent is free. With $\alpha =2$, it comes that $R^2$ remain constant or decrease by about 0.1. Given the uncertainties, we cannot conclude that the fit with exponent $\alpha$ set to 2 would be better, and that the initial growth process is independent of the culture medium. $\beta$ are found for respectively M2 and M0 culture media $2300\pm 200$, $5400\pm 400$, $2100\pm 100$ and $3300\pm 400$, $4900\pm 400$, $11{,}200\pm 2600$ h$^2$ mm$^{-2}$. The values of $\beta$ extracted in this fit are not found to be compatible and allow us to conclude that the first order effect on the initial growth is due to the linear rather than exponential law of the number of apexes *A*.

## Conclusion

In this work, we propose the simulation of the growth of the *P. anserina* branching network, based on a binary tree and whose parameters are finely improved from experimental observations. In particular, they take into account the separation of branching statistics of lateral and apical apexes, the spontaneous curvature of hyphae outside branching events, the average orientation of branches and aging effects due to extreme densification. This allows to construct an expression of the density based on the growth laws of the number of apexes *A* and a surface constructed with the eigenvalues of the matrix of apexes locations. A typical behavioral law on densification is then derived, which is expected to follow two phases, separated by a minimum, corresponding to a growth time $t_{\text {min}}$ whose expression depends only on the growth rate of the apexes and the degree of the polynomial expressing the variation of the occupied surface. The typical behavior is well found experimentally, as well as the dependence of the value of $t_{\text {min}}$ on the growth rate of *A*, and thus on the culture medium. The two phases of densification are explained by the difference in the lengths of the respective apical dominances of the apical and lateral branches. Moreover, a deviation from the typical law at the beginning of growth is observed and discussed. This behavior is compatible with a change observed in the growth dynamics of the number of apexes, being initially linear rather than exponential.

Finally, it appears that the density observable could be a judicious parameter for the characterization of a fungal thallus under constraints in further experiments (as for example here, the growth on a poor-nutrient medium).

## Data Availability

The data used in this work can be downloaded from a scientific data sharing site^32^.
